# Supplementary material for: Ultra-high precision nano additive manufacturing of metal oxide semiconductors via multi-photon lithography
Source: Nat Commun. 2024 Oct 25;15:9216. doi: 10.1038/s41467-024-52929-8 (PMC11511962; doi:10.1038/s41467-024-52929-8)
Supplement: Supplementary file 1 — Supplementary Information [file 41467_2024_52929_MOESM1_ESM.pdf]

Supplementary Information

Ultra-high Precision Nano Additive Manufacturing of Metal Oxide Semiconductors Via Multi-photon Lithography

Chun Cao,[1,#] Xianmeng Xia,[2,#] Xiaoming Shen,[3,#] Xiaobing Wang,[2] Zhenyao Yang,[2] Qiulan Liu,[4*] Chenliang Ding,[4] Dazhao Zhu,[4] Cuifang Kuang,[3,4*] and Xu Liu [3,4]

[1]School of Mechanical Engineering, Hangzhou Dianzi University, Hangzhou 310018, China

[2]Research Center for Astronomical Computing, Zhejiang Lab, Hangzhou 311121, China

[3]ZJU-Hangzhou Global Scientific and Technological Innovation Center, Hangzhou, 311200, China

[4]State Key Laboratory of Extreme Photonics and Instrumentation, College of Optical Science and Engineering, Zhejiang University, Hangzhou 310027, China

[#]These authors contributed equally: Chun Cao, Xianmeng Xia, Xiaomeng Shen

*Corresponding author: 21430066@zju.edu.cn, cfkuang@zju.edu.cn

Table of Contents

Section A: Supplementary Figures---S3

Section B: Supplementary Tables---S16

Section C: Supplementary References---S20

Section A. Supplementary Figures

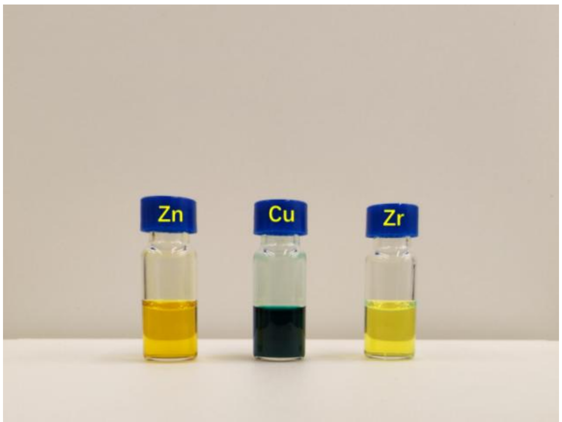

Supplementary Fig. 1 The images of the as-prepared photoresists. Zinc-based photoresist (left), copper-based photoresist (middle) and zirconium-based photoresist (right) before spin-coating.

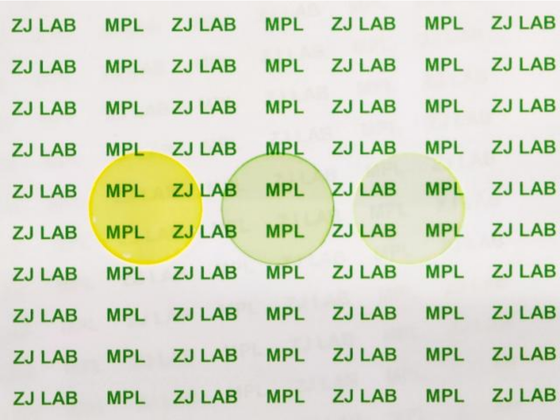

Supplementary Fig. 2 Thin films obtained by spin coating different photoresists. Zinc-based photoresist (left), copper-based photoresist (middle) and zirconium-based photoresist (right).

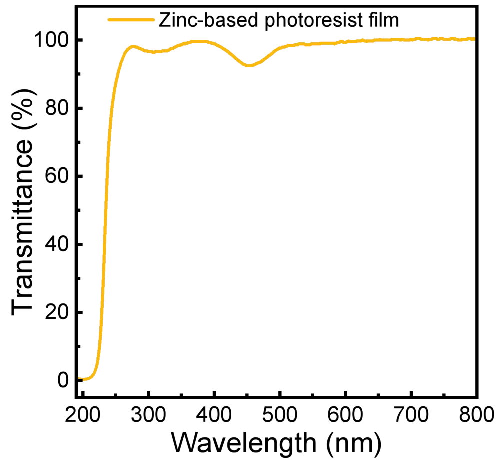

Supplementary Fig. 3 Optical transmittance curve of zinc-based photoresist. Spin-coated on a quartz substrate.

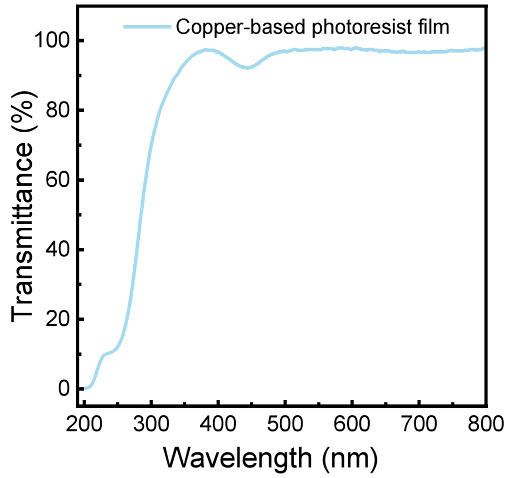

Supplementary Fig. 4 Optical transmittance curve of copper-based photoresist. Spin-coated on a quartz substrate.

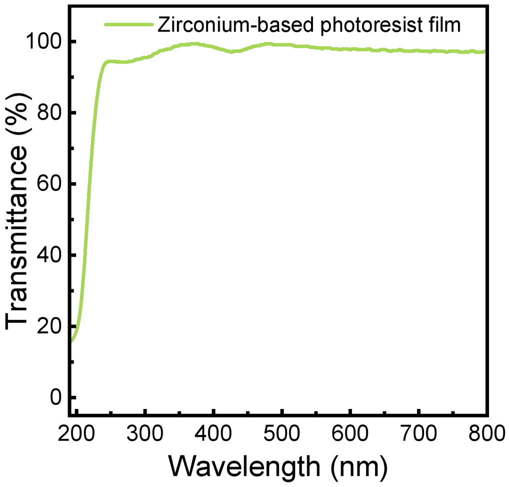

Supplementary Fig. 5 Optical transmittance curve of zirconium-based photoresist. Spin-coated on a quartz substrate.

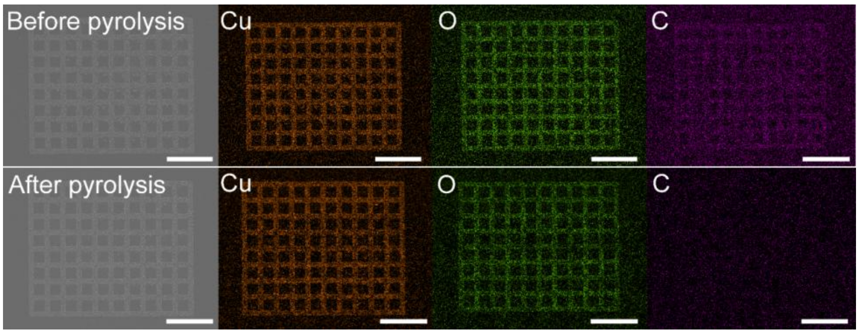

Supplementary Fig. 6 SEM images and EDS maps of copper-based photoresist. The grid patterns are fabricated at 25 mW and 2 mm s^{-1}. scale bar: 25 μm. The pre-pyrolysis pattern mainly contains Cu, O and C elements, and it is difficult to distinguish C elements in post-pyrolysis pattern, indicating that the organic components were removed during the pyrolysis process.

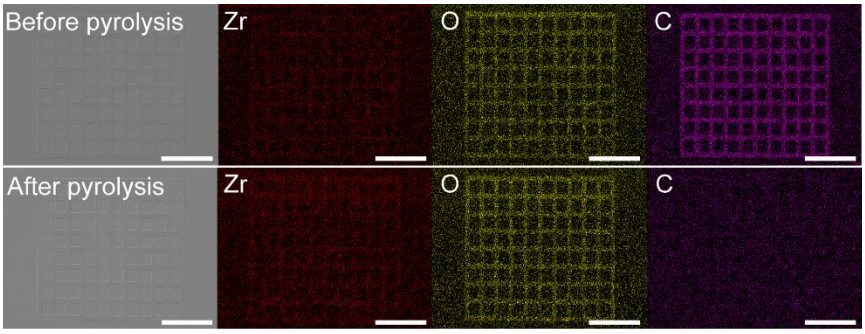

Supplementary Fig. 7 SEM images and EDS maps of zirconium-based photoresist. The grid patterns are fabricated at 25 mW and 5 mm s^{-1}. scale bar: 25 μm. The pre-pyrolysis pattern mainly contains Zr, O and C elements. The contrast of zirconium map image is low because the excitation energy of zirconium is very close to that of platinum (sputtered conductive layer), but the pattern can still be distinguished. And it is difficult to distinguish C elements in post-pyrolysis pattern, indicating that the organic components were removed during the pyrolysis process.

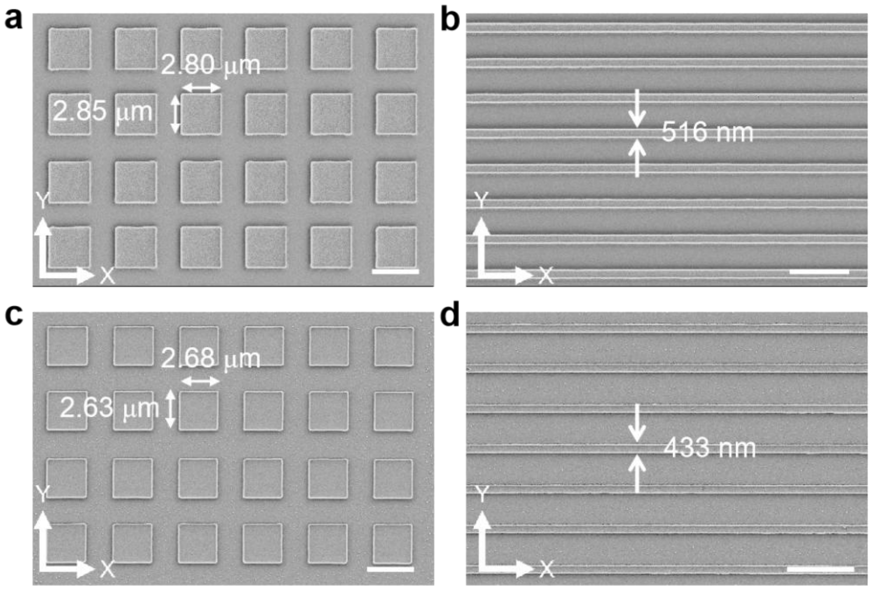

Supplementary Fig. 8 SEM images of patterns fabricated using zinc-based photoresist. **a-b** Before pyrolysis. **c-d** After pyrolysis. Rectangular blocks fabricated at 25 mW and 2 mm s^{-1}. Scale bar: 3 μm.

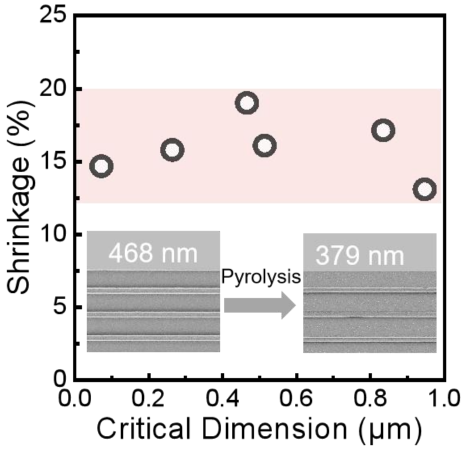

Supplementary Fig. 9 The shrinkage rate of the patterns with a CD below 1 μm. The insert shows a typical line before and after pyrolysis.

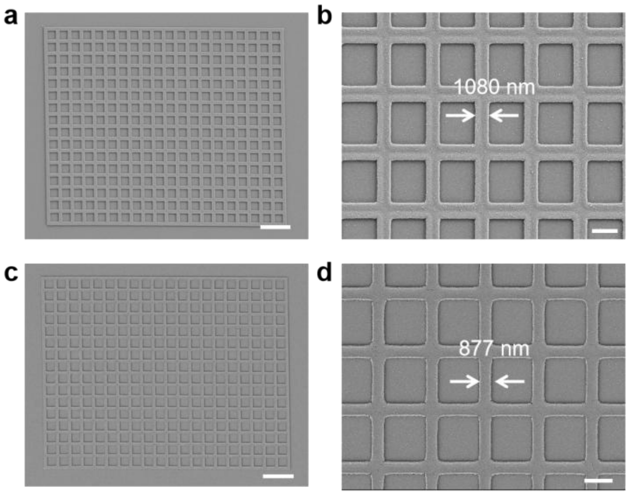

Supplementary Fig. 10 SEM images of patterns fabricated using zinc-based photoresist. a-b Before pyrolysis. **c-d** After pyrolysis. The grid pattern are fabricated at 25 mW and 2 mm s^{-1}. Scale bar in **a** and **c** is 10 μm, and scale bar in **b** and **d** is 2 μm

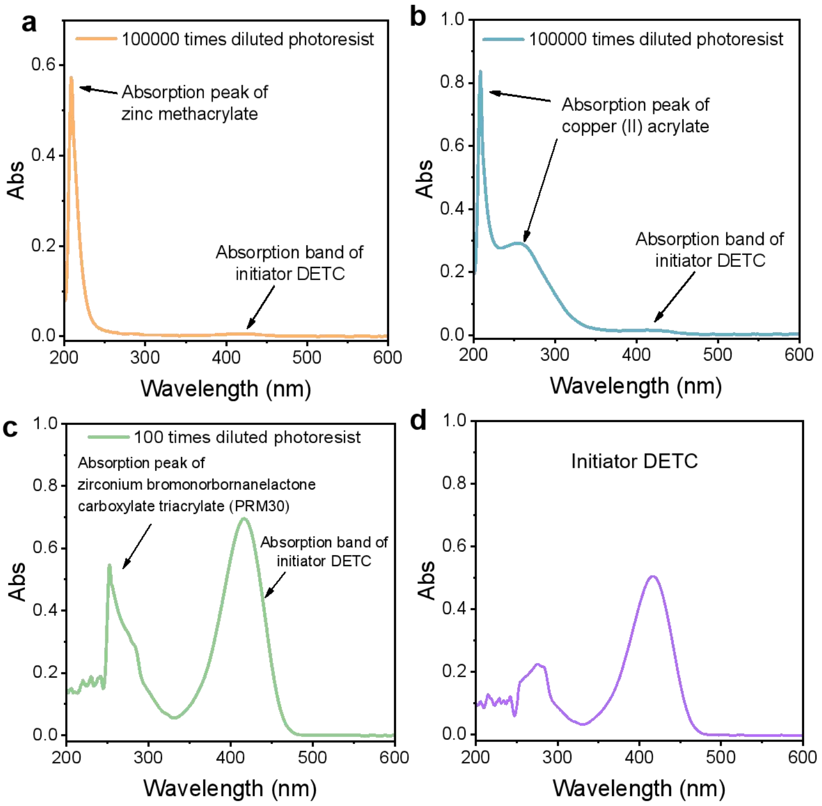

Supplementary Fig. 11 The absorption spectrum of different photoresists. a zinc-based, **b** copper-based, **c** zirconium-based photoresist and **d** initiator DETC.

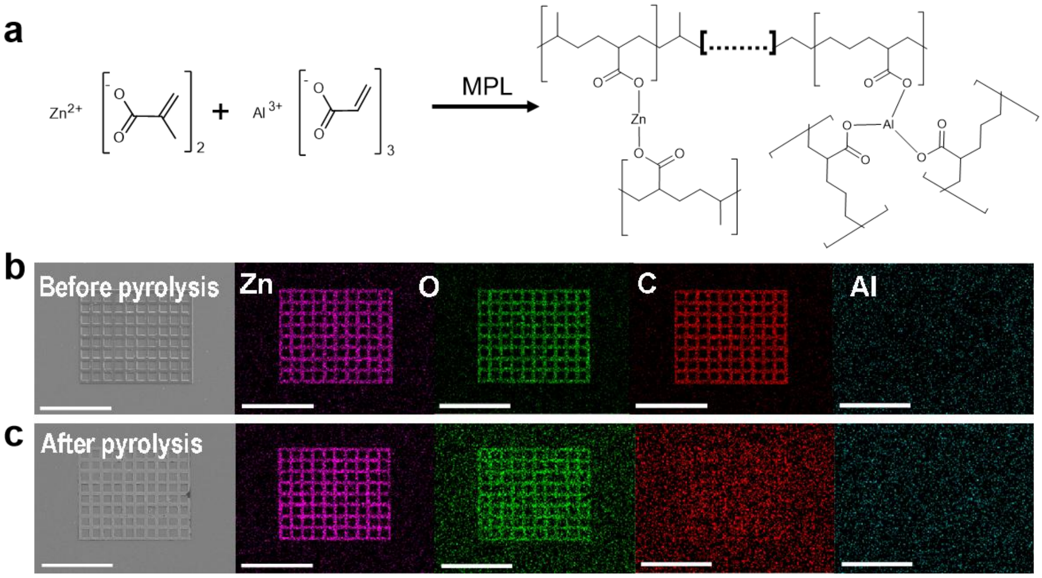

Supplementary Fig. 12 Preparation of Al doped ZnO by MPL. a Schematic diagram of the principle of Al doping. SEM images and EDS maps of Al doped zinc-based photoresists **b** before pyrolysis and **c** after pyrolysis. The grid patterns are fabricated at 25 mW and 2 mm s^{-1}. Scale bar: 50 μm.

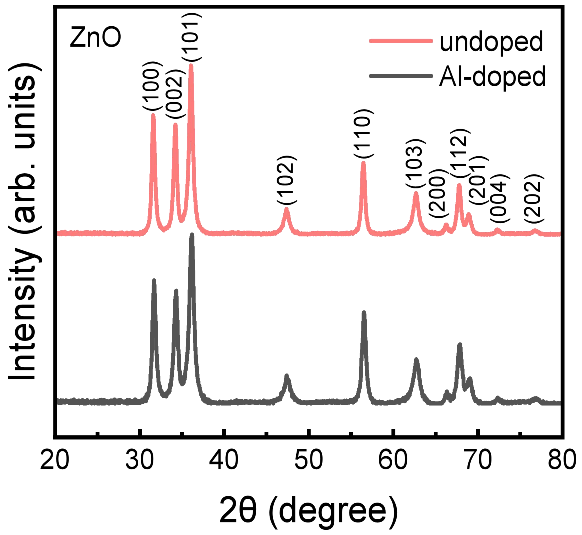

Supplementary Fig. 13 XRD spectra of ZnO and Al-doped ZnO by our strategy.

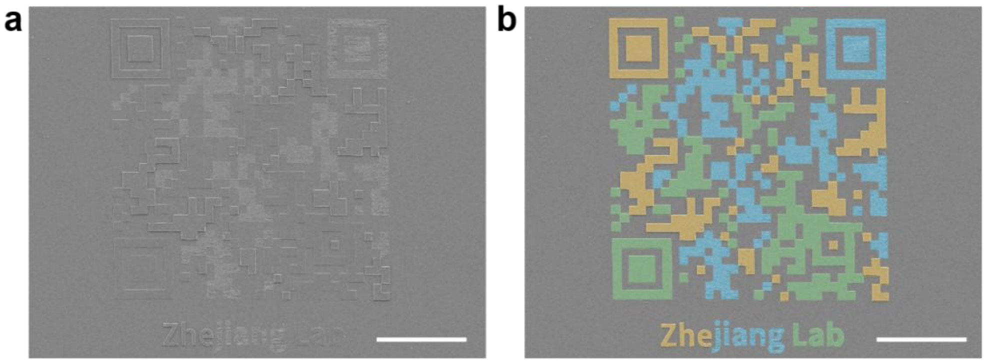

Supplementary Fig. 14 SEM image of a QR code pattern. a is the original image. **b** is the colored image to distinguish the different MOS. ZnO (yellow part) fabricated at 25 mW and 2 mm s^{-1}, CuO (blue part) fabricated at 20 mW and 3 mm s^{-1}, ZrO_2 (green part) fabricated at 20 mW and 10 mm s^{-1}. Scale bar: 25 μm.

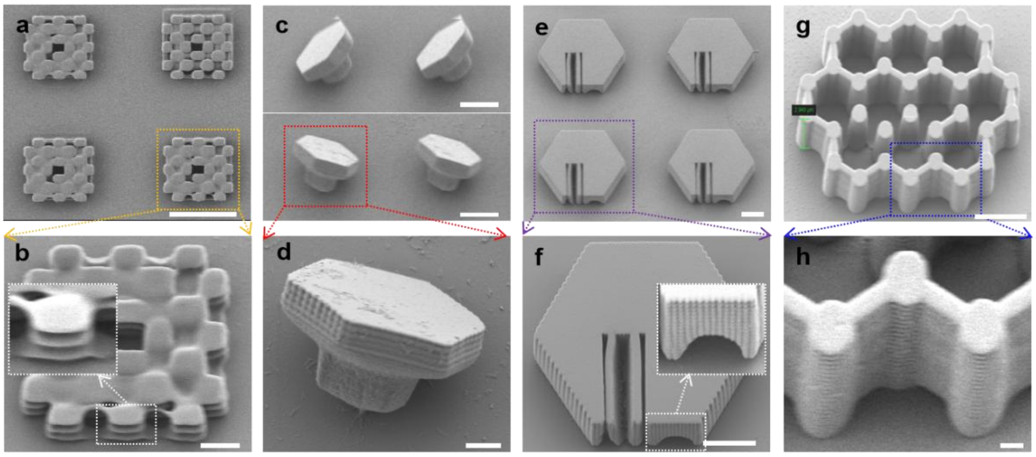

Supplementary Fig. 15 3D micro-structures fabricated by our strategy using zirconium-based photoresist. a-b Bolt arrays (the thread on the side can be clearly seen). **c-d** Bolt bases. **e-f** Hexagonal columns with channels. **g-h** Cell fence. Scale bar in **a**, **c**, **e**, **f** and **g** is 5 μm, scale bar in **b**, **d** and **h** is 1μm.

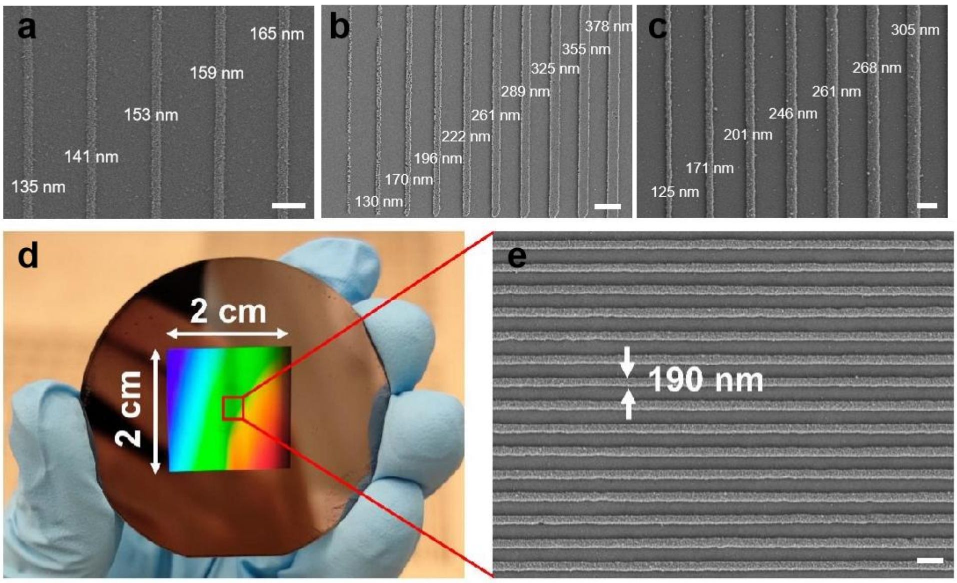

Supplementary Fig. 16 MPL of solid precursor photoresist using air objectives (50x, NA=0.95). **a** SEM images of lines written using Zn-based precursor photoresist, the laser power from left to right are 14.8 mW, 15.5 mW, 16.1 mW, 16.7 mW and 17.3 mw, respectively. The writing speed is constant at 10 mm s^{-1}. **b** SEM images of lines written using Cu-based precursor photoresist, the laser power from left to right are 8.2 mW, 8.7 mW, 9.2 mW, 9.7 mW, 10.2 mW, 10.8 mW, 11.3 mW, 11.9 mW and 12.5 mW,

respectively. The writing speed is constant at 1 mm s^{-1}. **c** SEM images of lines written using Zr-based precursor photoresist, the laser power from left to right are 6.3 mW, 6.7 mW, 7.2 mW, 7.7 mW, 8.2 mW and 8.7 mW, respectively. The writing speed is constant at 10 mm s^{-1}. **d** A 2 cm x 2 cm grating with a linewidth of 190 nm. **e** The line array (500 nm in resolution) written using Zr-based precursor photoresist at 13.1 mW and 100 mm s^{-1}. Scale bar: 500 nm.

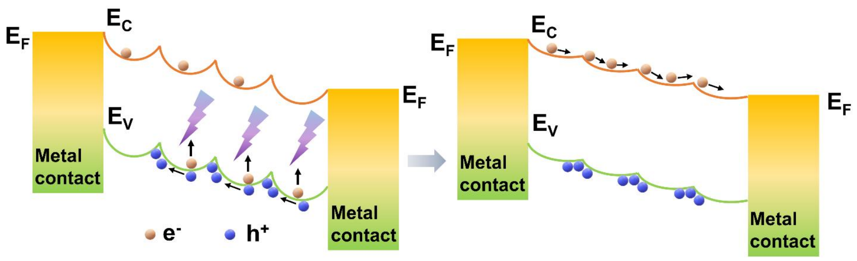

Supplementary Fig. 17 Schematics of carrier transport mechanism in ZnO photodetector under UV illumination. For polycrystalline ZnO, the grain boundary is equivalent to the energy barrier of carrier transport in dark condition. And under UV illumination, excessive photogenic holes will migrate to the grain boundary driven by the established internal electric field, resulting in lower barrier height[1].

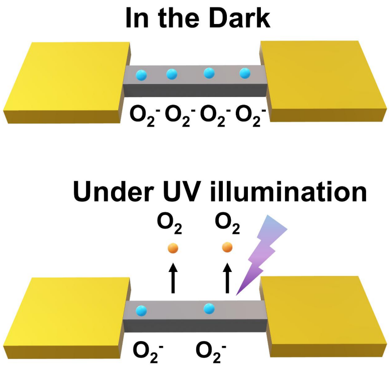

Supplementary Fig. 18 Photoconductive gain caused by the desorption of oxygen on the ZnO surface under UV illumination. It is common accepted that the photoconductance gain of ZnO is due to the photoelectric effect and oxygen desorption at the interface. When exposed to UV light, the reaction on the surface of ZnO can be summarized as follows:

$$O_{2(g)} + e^- \rightarrow O^-_{2(ad)} \qquad (1)$$

$$hv \rightarrow e^- + h^+ \qquad (2)$$

$$h^+ + O^-_{2(ad)} \rightarrow O_{2(g)} \qquad (3)$$

UV light excites electrons to produce photogenerated electron-hole pairs (Equation 2), and the holes are easy to migrate to the surface to be trapped, resulting in the desorption of oxygen, which further reduces the consumption of electrons. Once external bias is applied, the extra electrons cause the gain of photocurrent [2, 3].

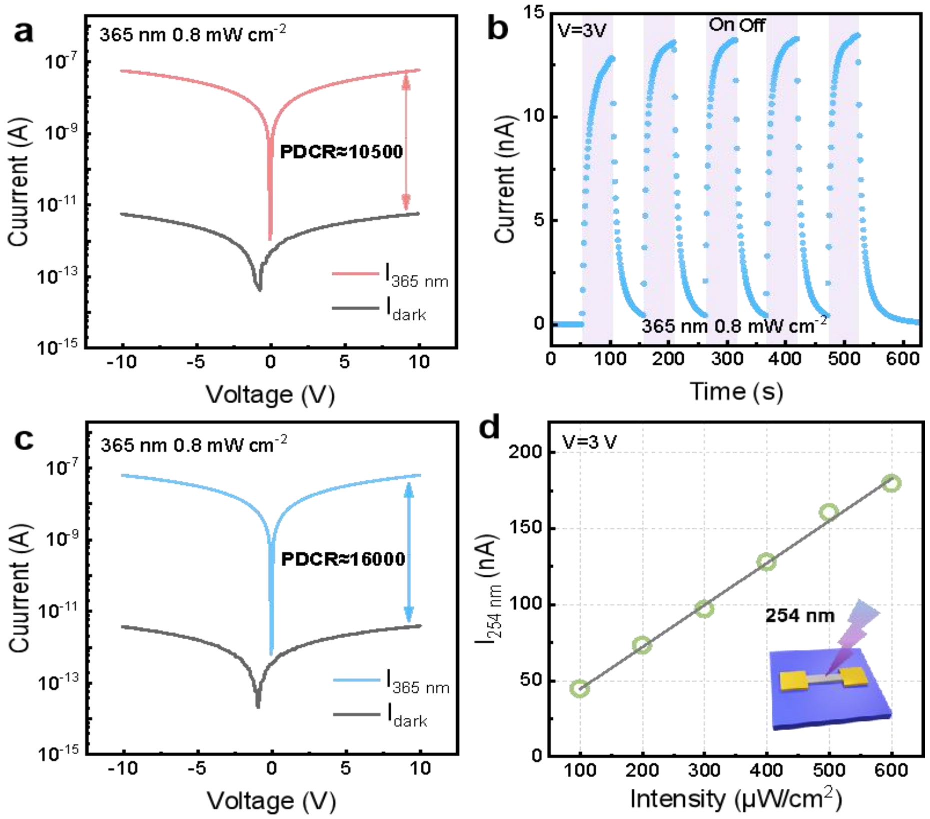

Supplementary Fig. 19 Performances of ZnO and Al-doped ZnO UV photodetector. a I–V characteristic curves of ZnO UV photodetector under 365 nm UV illumination (0.8 mW cm^{-2}). **b** Time-dependent photoresponse of Al-doped ZnO UV photodetector under a pulsed 365 nm UV illumination (0.8 mW cm^{-2}) with 3V external bias. **c** I–V characteristic curves of Al-doped ZnO UV photodetector under 365 nm UV illumination (0.8 mW cm^{-2}). **d** Current response of ZnO UV photodetector under different intensity of 254 nm UV light (3 V bias).

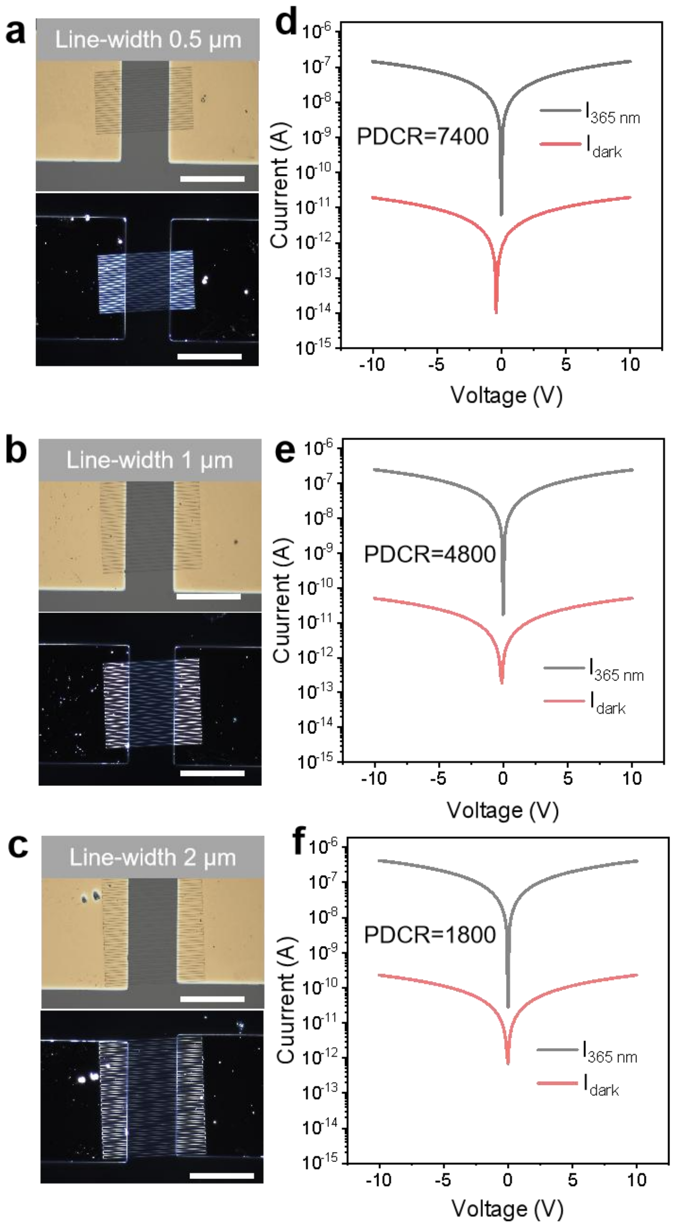

Supplementary Fig. 20 Performances of ZnO UV photodetector with various line widths. Optical microscope images (up: open-field mode, down: dark-field mode) of ZnO UV photodetector with different line-widths: **a** 0.5 μm, **b** 1 μm, **c** 2 μm. I–V characteristic curves of ZnO UV photodetector in the dark and under 365 nm UV illumination (0.2 mW cm^{-2}): **d** 0.5 μm, **e** 1 μm, **f** 2 μm. PDCR means photo-to-dark current ratio. Scale bar: 50 μm.

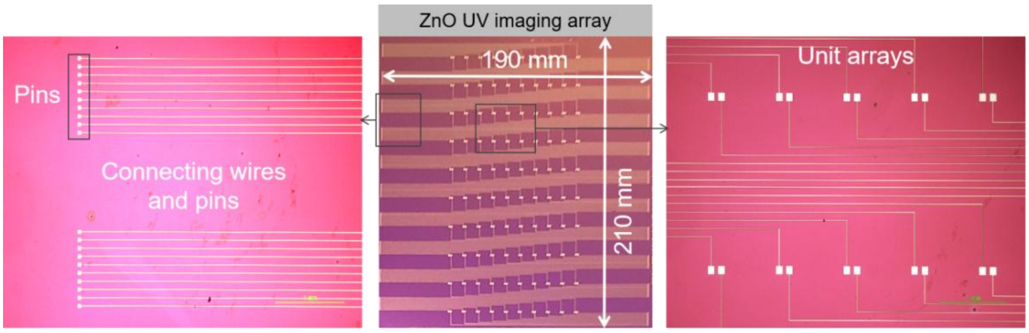

Supplementary Fig. 21 The images of ZnO UV imaging arrays and its details. Pins for sensing performance testing via probes.

Section B. Supplementary Tables

Supplementary Table 1

Theoretical mass ratios of monomers, metal elements and metal oxides to initial photoresist films

	zinc-based photoresist	copper-based photoresist	zirconium-based photoresist
Monomer (wt%)	98.36	99.01	99.01
Metallic elements (wt%)	27.31	30.59	15.05
Metal oxides (wt%)	33.99	38.30	20.33
Mass reduction (wt%)	66.01	61.70	79.67

Supplementary Table 2

Comparison of minimum characteristic dimension of metal oxides additive manufacturing technology[4-20].

#	Groups	Technologies	Materials	Critical dimension, (μm)	Ref
1	Farandos et al., 2016	Inkjet printing	Yttria-stabilized zirconia (YSZ)	35	[4]
2	Li et al., 2022	Digital light process (DLP)	CuO, NiO, etc.	28	[5]
3	Kim et al., 2016	Electrohydrodynamic (EHD) Inkjet Printing	In_2O_3	2	[6]
4	Zhang et al., 1999	Micro-stereolithography (μSL)	Al_2O_3	1.2	[7]
5	Yu et al., 2018	Two-Photon lithography (TPL)	TiO_2	0.65	[8]
6	Passinger et al., 2007	TPL	TiO_2	0.4	[9]
7	Cho et al., 2020	area-selective atomic layer deposition (AS-ALD)	ZnO, Al_2O_3, SnO_2	0.312	[10]
8	Yang et al., 2023	Laser-induced hydrothermal synthesis	ZnO	0.26	[11]
9	Yee et al., 2019	TPL	ZnO	0.25	[12]
10	Long et al., 2020	TPL	ZnO (nanowires)	0.24	[13]
11	Liu et al., 2021	TPL	ZnO, Co_3O_4	0.17	[14]
12	Guo et al., 2010	TPL	SnO_2	0.15	[15]
13	Gailevičius et al., 2018	TPL	ZrO_2	0.1	[16]
14	Desponds et al., 2021	TPL	ZrO_2	0.1	[17]
15	Malinauskas et al., 2022	TPL	SiO_2/ZrO_2	0.06	[18]
16	This work	MPL	ZnO, CuO, ZrO_2	0.035	
17	Auzelyte et al., 2010	Extreme ultra-violet interference lithography (EUV-IL)	ZnO	0.01	[19]
18	Saifullah et al., 2005	Electron Beam Lithography (EBL)	ZnO	0.005	[20]

Supplementary Table 3

Excited-state energies and ionization potentials of the molecules under investigation determined numerically using the PBE1PBE / Def2TZVP method. The corresponding number of excitation photons (2.36 eV) with the same energy is also given.

	S_0-S_1 energy (ev)	Corresponding number of photons	Ionization energy (ev)	Corresponding number of photons
Zinc methacrylate	5.2	2.2	9.4	4.0
Copper acrylate	2.0	0.9	9.5	4.0
Zirconium bromonorbornanelactone carboxylate triacrylate (PRM30)	4.3	1.8	8.8	3.7

Supplementary Table 4

The performance comparison of ZnO UV photodetectors[21-50].

Authors	Types of MOS	PDCR value	Journal, year	Ref.
Basavaraj G. Hunashimarad, et al.	Ca-doped ZnO film	3.17 at 365 nm	Optical Materials 2022	[21]
Sabina M. Hatch, et al.	ZnO-nanorods-Cu SCN	4.5 at 375 nm	Advanced materials 2013	[22]
Shoou-Jinn Chang, et al.	Fe-doped ZnO	<10 at 375 nm	IEEE Photonics Technology Let. 2013	[23]
Qi Li, et al.	ZnO-CuO nanorod	10 at 325 nm	Advanced optical materials 2022	[24]
Chih-Hung Hsiao, et al.	Needle-like Ga-ZnO nanorods	11.07 at 360 nm	IEEE transactions on electron devices 2013	[25]
Jing Wang, et al.	ZnO nanowires	24.2 at 365 nm	J. Mater. Chem. C 2016	[26]
Chiung-Hsien Huang, et al.	Li-doped ZnO nanorods	34.87 at 380 nm	Microsystem Technologies 2022	[27]
Sunghoon Park, et al.	ZnO nanowires	49 at 365 nm	Journal of Alloys and Compounds 2016	[28]
Min Chen, et al.	ZnO hollow-sphere nanofilm	53 at 350 nm	Small 2011	[29]
Huihui Yu, et al.	Atomic-thin ZnO Sheet	69.6 ay 365 nm<br>120.1 at 254 nm	Small 2020	[30]
Cheng-Liang, Hsu et al.	Vertical ZnO nanowires	67.5 at 254 nm	Chemical Physics Letters 2005	[31]
Soo Hyun Lee, et al.	ZnO nanorods	1720 at 380 nm	Nanoscale Research Letters 2016	[32]
Shaivalini Singh, et al.	Al-doped ZnO	3327.94 at 372 nm	MicrosystemTechnologies 2016	[33]
Nishant Kumar, et al.	Mg-doped ZnO films	71.68 at 365 nm	Journal of Alloys and Compounds 2018	[34]
Nishant Kumar, et al.	Cd-doped ZnO	93.78 at 386 nm	Journal of Alloys and Compounds 2017	[35]
S.J. Young, et al.	ZnO film	290 at 370 nm	Sensors and Actuators A: Physical 2007	[36]
Yen-Lin Chu, et al.	Ni-doped ZnO	393.04 at 380nm	J. Electrochem. Soc. 2020	[37]
Fatemeh Abbasi, et al.	Ni-doped ZnO film	416.14 at 350 nm	Optics Communications 2021	[38]
Ramazanali Dalvand, et al.	ZnO nanoneedles	600 at 325nm	Journal of Materials Science: Materials in Electronics 2018	[39]

Liu kw, et al.	Mg-doped ZnO film	Approx. 1000 at 368 nm	Sensors 2010	[40]
Akshta Rajan, et al.	ZnO thin film	1000 at 365 nm	MRS Online Proceedings Library 2013	[41]
Hsiang-Chun Wang, et al.	Ag nanoparticles modified ZnO	1000 at 365 nm	Nanoscale Research Letters 2020	[42]
Zeping Li, et al.	ZnO quantum dot	1767.8 at 350 nm	Applied Surface Science 2022	[43]
Jingwei Liu, et al.	ZnO nanowire	3000 at 365	Adv. Mater. Technol. 2022	[44]
James Taban Abdalla, et al.	ZnO nanorod	4000 at 365 nm	Journal of Electronic Materials 2020	[45]
Fa Cao, et al.	ZnO-CuI heterostructure	4250 at 365nm	Journal of Alloys and Compounds 2021	[46]
Dawit Gedamuv, et al.	ZnO nanotetrapod networks	4500 at 365 nm	Advanced Materials 2014	[47]
Omar F. Farhat, et al.	ZnO nanoaggregates	8345 at 365 nm	Sensors and Actuators A: Physical 2021	[48]
Amit Kumar Rana, et al.	Co3O4-ZnO film	45700 at 365 nm	Materials Science in Semiconductor Processing 2020	[49]
Jin Hyung Jun, et al.	ZnO nanoparticles	10^6 at 325 nm	Ceramics International 2009	[50]
ZnO wire		10500 at 365 nm	This work	
Al-doped ZnO wire		16000 at 365 nm	This work	
ZnO wire		100000 at 254 nm	This work	

Section C. Supplementary References

1. Liu, X. et al. All-printable band-edge modulated ZnO nanowire photodetectors with ultra-high detectivity. *Nat. Commun.* **5**, 4007 (2014).
2. Soci, C., et al. ZnO nanowire UV photodetectors with high internal gain. *Nano Lett.* **7**, 1003-1009 (2007).
3. Yeo, J., et al. Rapid, One-Step, Digital Selective Growth of ZnO Nanowires on 3D Structures Using Laser Induced Hydrothermal Growth. *Adv. Funct. Mater.* **23**, 3316-3323 (2013).
4. Farandos, N. M., Kleiminger, L., Li, T., Hankin, A. & Kelsall, G. H. Three-dimensional Inkjet Printed Solid Oxide Electrochemical Reactors. I. Yttria-stabilized Zirconia Electrolyte. *Electrochim. Acta* **213**, 324-331 (2016).
5. Li, Y., et al. Incorporating Metal Precursors towards a Library of High-resolution Metal Parts by Stereolithography. *Appl. Mater. Today* **29**, 101553 (2022).
6. Kim, S. Y., et al. High-resolution electrohydrodynamic inkjet printing of stretchable metal oxide semiconductor transistors with high performance. *Nanoscale* **8**, 17113-17121 (2016).
7. Zhang, X., Jiang, X. N. & Sun, C. Micro-stereolithography of polymeric and ceramic microstructures. *Sensor. Actuat. A-Phys.* **77**, 149-156 (1999).
8. Yu, S. Y., et al. Direct Laser Writing of Crystallized TiO_2 and TiO_2/Carbon Microstructures with Tunable Conductive Properties. *Adv. Mater.* **30**, 1805093 (2018).
9. Passinger, S., et al. Direct 3D Patterning of TiO_2 Using Femtosecond Laser Pulses. *Adv. Mater.* **19**, 1218-1221 (2007).
10. Cho, T. H., et al. Area-Selective Atomic Layer Deposition Patterned by Electrohydrodynamic Jet Printing for Additive Manufacturing of Functional Materials and Devices. *ACS Nano* **14**, 17262-17272 (2020).
11. Yang, L., et al. Laser printed microelectronics. *Nat. Commun.* **14**, 1103 (2023).
12. Yee, D. W., Lifson, M. L., Edwards, B. W. & Greer, J. R. Additive Manufacturing of 3D-Architected Multifunctional Metal Oxides. *Adv. Mater.* **31**, 1901345 (2019).
13. Long, J., et al. Directional Assembly of ZnO Nanowires via

Three-Dimensional Laser Direct Writing. *Nano Lett.* **20**, 5159-5166 (2020).

14. Liu, J., et al. 3D Printing Nano‐Architected Semiconductors Based on Versatile and Customizable Metal‐Bound Composite Photoresins. *Adv. Mater. Technol.* **7**, 2101230 (2022).
15. Guo, L., et al. Femtosecond laser direct patterning of sensing materials toward flexible integration of micronanosensors. *Opt. Lett.* **35**, 1695-1697 (2010).
16. Gailevičius, D., et al. Additive-manufacturing of 3D glass-ceramics down to nanoscale resolution. *Nanoscale Horiz.* **4**, 647-651 (2019).
17. Desponds, A., et al. 3D Printing and Pyrolysis of Optical ZrO_2 Nanostructures by Two-Photon Lithography: Reduced Shrinkage and Crystallization Mediated by Nanoparticles Seeds. *Small* **17**, 2102486 (2021).
18. Merkininkaitė, G., Aleksandravičius, E., Malinauskas, M., Gailevičius, D. & Šakirzanovas, S. Laser additive manufacturing of Si/ZrO_2 tunable crystalline phase 3D nanostructures. *Opto-Electronic Adv.* **5**, 210077 (2022).
19. Auzelyte, V., Sigg, H., Schmitt, B. & Solak, H. H. Direct formation of ZnO nanostructures by chemical solution deposition and EUV exposure. *Nanotechnology* **21**, 215302 (2010).
20. Saifullah, M. S. M., et al. Sub-10 nm High-Aspect-Ratio Patterning of ZnO Using an Electron Beam. *Adv. Mater.* **17**, 1757-1761 (2005).
21. Hunashimarad, B. G., Bhat, J. S., Raghavendra, P. V. & Bhajantri, R. F. ZnO:Ca MSM ultraviolet photodetectors. *Opt. Mater.* **124**, 111960 (2022).
22. Hatch, S. M., Briscoe, J. & Dunn, S. A Self-Powered ZnO-Nanorod/CuSCN UV Photodetector Exhibiting Rapid Response. *Adv. Mater.* **25**, 867-871 (2013).
23. Chang, S. J., et al. Noise Properties of Fe-ZnO Nanorod Ultraviolet Photodetectors. *IEEE Photonic. Tech. L.* **25**, 2089-2092 (2013).
24. Li, Q., Huang, J., Meng, J. & Li, Z. Enhanced Performance of a Self-Powered ZnO Photodetector by Coupling LSPR-Inspired Pyro-Phototronic Effect and Piezo-Phototronic Effect. *Adv. Opt. Mater.* **10**, 2102468 (2022).
25. Hsiao, C. H., et al. Field-Emission and Photoelectrical Characteristics of

Ga-ZnO Nanorods Photodetector. *IEEE T. Electron Dev.* **60**, 1905-1910 (2013).

26. Wang, J., et al. Ligand-directed rapid formation of ultralong ZnO nanowires by oriented attachment for UV photodetectors. *J. Mater. Chem. C* **4**, 5755-5765 (2016).
27. Huang, C. H., et al. Fabrication and characterization of homostructured photodiodes with Li-doped ZnO nanorods. *Microsyst. Technol.* **28**, 369-375 (2022).
28. Park, S., et al. ZnO-core/ZnSe-shell nanowire UV photodetector. *J. Alloy. Compound.* **658**, 459-464 (2016).
29. Chen, M., et al. ZnO Hollow-Sphere Nanofilm-Based High-Performance and Low-Cost Photodetector. *Small* **7**, 2449-2453 (2011).
30. Yu, H., et al. Atomic-Thin ZnO Sheet for Visible-Blind Ultraviolet Photodetection. *Small* **16**, 2005520 (2020).
31. Hsu, C. L., et al. Ultraviolet photodetectors with low temperature synthesized vertical ZnO nanowires. *Chem. Phys. Lett.* **416**, 75-78 (2005).
32. Lee, S. H., Kim, S. H. & Yu, J. S. Metal-Semiconductor-Metal Near-Ultraviolet (~380 nm) Photodetectors by Selective Area Growth of ZnO Nanorods and SiO_2 Passivation. *Nanoscale Res. Lett.* **11**, 333 (2016).
33. Singh, S. Al doped ZnO based MISIM ultraviolet photodetectors. *Microsystem Technol.* **23**, 999-1003 (2017).
34. Kumar, N. & Srivastava, A. Green photoluminescence and photoconductivity from screen-printed Mg doped ZnO films. *J. Alloy. Compound.* **735**, 312-318 (2018).
35. Kumar, N. & Srivastava, A. Faster photoresponse, enhanced photosensitivity and photoluminescence in nanocrystalline ZnO films suitably doped by Cd. *J. Alloy. Compound.* **706**, 438-446 (2017).
36. Young, S. J., et al. ZnO-based MIS photodetectors. *Sensor. Actuat. A-Phys.* **135**, 529-533 (2007).
37. Chu, Y. L., et al. Fabrication and Characterization of Ni-Doped ZnO Nanorod Arrays for UV Photodetector Application. *J. Electrochem. Soc.* **167**, 067506 (2020).
38. Abbasi, F., Zahedi, F. & Yousefi, M. h. Fabricating and investigating high photoresponse UV photodetector based on Ni-doped ZnO nanostructures.

Opt. Commun. **482**, 126565 (2021).

39. Dalvand, R., Mahmud, S. & Shabannia, R. Fabrication of UV photodetector using needle-shaped ZnO nanostructure arrays prepared on porous silicon substrate by a facile low-temperature method. *J. Mater. Sci-Mater. El.* **29**, 4999-5008 (2018).
40. Liu, K., Sakurai, M. & Aono, M. ZnO-Based Ultraviolet Photodetectors. *Sensors* **10**, 8604-8634 (2010).
41. Rajan, A., Paliwal, A., Gupta, V. & Tomar, M. Plasmonic Enhancement of Optical Absorption of UV Radiation in ZnO Thin Film Based Ultraviolet Photodetectors. *MRS Online Proceedings Library* **1509**, 1212 (2013).
42. Wang, H. C., et al. ZnO UV Photodetectors Modified by Ag Nanoparticles Using All-Inkjet-Printing. *Nanoscale Res. Lett.* **15**, 176 (2020).
43. Li, Z., et al. High performance ZnO quantum dot (QD)/ magnetron sputtered ZnO homojunction ultraviolet photodetectors. *Appl. Surf. Sci.* **582**, 152352 (2022).
44. Liu, J., et al. 3D Printing Nano-Architected Semiconductors Based on Versatile and Customizable Metal-Bound Composite Photoresins. *Adv. Mater. Technol.* **7**, 2101230 (2022).
45. Abdalla, J. T., et al. Enhanced Ag@SnO_2 Plasmonic Nanoparticles for Boosting Photoluminescence and Photocurrent Response of ZnO Nanorod UV Photodetectors. *J. Electron. Mater.* **49**, 5657-5665 (2020).
46. Cao, F., Jin, L., Wu, Y. & Ji, X. High-performance, self-powered UV photodetector based on Au nanoparticles decorated ZnO/CuI heterostructure. *J. Alloy. Compound.* **859**, 158383 (2021).
47. Gedamu, D., et al. Rapid Fabrication Technique for Interpenetrated ZnO Nanotetrapod Networks for Fast UV Sensors. *Adv. Mater.* **26**, 1541-1550 (2014).
48. Farhat OF, Husham M, Bououdina M, Abuelsamen AA, Oglat AA, Mohammed NJ. Tape-based novel ZnO nanoaggregates photodetector. Sensors and Actuators A: Physical 332, 113210 (2021).
49. Rana, A. K., Patel, M., Nguyen, T. T., Yun, J. H. & Kim, J. Transparent Co_3O_4/ZnO photovoltaic broadband photodetector. *Mat. Sci. Semicon. Proc.* **117**, 105192 (2020).
50. Jun, J. H., Seong, H., Cho, K., Moon, B. M. & Kim, S. Ultraviolet

photodetectors based on ZnO nanoparticles. *Ceram. Int.* **35**, 2797-2801 (2009).
